# Peripheral Blood Circular RNAs as a Biomarker for Major Depressive Disorder and Prediction of Possible Pathways

**DOI:** 10.3389/fnins.2022.844422

**Published:** 2022-03-31

**Authors:** Dandan Zhang, Yao Ji, Xiongjin Chen, RunSen Chen, Yaxue Wei, Qian Peng, Juda Lin, Jingwen Yin, Hezhan Li, Lili Cui, Zhixiong Lin, Yujie Cai

**Affiliations:** ^1^Guangdong Key Laboratory of Age-Related Cardiac and Cerebral Diseases, Affiliated Hospital of Guangdong Medical University, Zhanjiang, China; ^2^Department of Psychiatry, Affiliated Hospital of Guangdong Medical University, Zhanjiang, China; ^3^Department of Rehabilitation Medicine Guangzhou Red Cross Hospital Affiliated to Jinan University, Guangzhou, China; ^4^School of Humanities and Management, Guangdong Medical University, Dongguan, China

**Keywords:** major depressive disorder, circular RNA, biomarker, neuroplasticity, bioinformatics analysis

## Abstract

Circular RNAs (circRNAs) are highly expressed in the central nervous system and have been reported to be associated with neuropsychiatric diseases, but their potential role in major depressive disorder (MDD) remains unclear. Here, we demonstrated that there was a disorder of circRNAs in the blood of MDD patients. It has been preliminarily proved that hsa_circ_0002473, hsa_circ_0079651, hsa_circ_0137187, hsa_circ_0006010, and hsa_circ_0113010 were highly expressed in MDD patients and can be used as diagnostic markers for MDD. Bioinformatics analysis revealed that hsa_circ_0079651, hsa_circ_0137187, hsa_circ_0006010, and hsa_circ_0113010 may affect the neuroplasticity of MDD through the ceRNA mechanism.

## Introduction

Depressive disorder is a set of diseases characterized by low spirit and there are approximately 264 million people worldwide suffer from it (GBD 2017 Disease and Injury Incidence and Prevalence Collaborators, 2018). According to DSM-5, depressive disorder includes major depressive disorder (MDD), persistent depressive disorder (PDD), premenstrual dysphoric disorder, disruptive mood dysregulation disorder, depressive disorder due to another medical condition, substance/medication-induced depressive disorder, etc. MDD is the most common type of depressive disorder, characterized by low spirits, loss of interest, inability to feel pleasure, poor concentration, etc. Relapse is also common, and to make matters worse, the patient may act or think suicidally. In comparison to men, women are more likely to suffer from major depression. Data from the China Mental Health Survey (CMHS), from 2013 to 2015, showed a lifetime prevalence of 3.4%, 12-month prevalence of 2.1%, and the 12-month prevalence is higher in women than in men (2.5 vs. 1.7%, *p* = 0.0061) (Huang et al., 2019). The etiology and pathogenesis of MDD remain unclear. Personality traits and environmental stress, as well as genetics, are thought to play a role in depression (Gonda et al., 2019). Some researchers believe that epigenetic gene modification and immune inflammation are linked to the development of MDD (Park et al., 2019; Beurel et al., 2020). The neurogenic theory posits that MDD is associated with neurogenesis defects, and postmortem investigations indicated that the size and density of neurons in the dorsolateral prefrontal cortex (dlPFC) and dentate gyrus (DG) of MDD patients were lower (Rajkowska, 2000; Boldrini et al., 2013; Malhi and Mann, 2018). However, there is currently no objective method to diagnose MDD. The diagnosis of MDD mainly depends on the patient’s subjective expression, which may be affected by the ability of both the patients and the clinicians to interpret symptoms. Further research into the pathogenesis of MDD will help to identify objective and reliable biomarkers of the disease, which can then be used in order to diagnose MDD, identify specific depressions, and select appropriate treatment.

CircRNAs are a class of covalently closed non-coding RNAs without 3′ poly-A tails or 5′ caps, characterized by high stability, wide expression, and tissue/developmental stage-specific expression, and are highly expressed in blood and brain (Rybak-Wolf et al., 2015). Circular RNAs are more stable and have a longer half-life than linear RNAs, and they are resistant to Ribonuclease R (RNase R) degradation, making them suitable as biomarkers for illness (Wen et al., 2020). CircRNAs have been found to have effects on neurological systems, innate immunity, microRNAs, and various disease-related pathways (Mei et al., 2019), and they are also linked to several neurological illnesses, including Alzheimer’s, Parkinson’s, multiple sclerosis, and schizophrenia (Gokool et al., 2020; Mehta et al., 2020). The field of circRNAs research in depression, however, is still in its infancy, with a few studies looking into the association between MDD and circRNAs (Cui et al., 2016; Zhang et al., 2018, 2019; Huang et al., 2020; Shi et al., 2021). Further studies are needed to fully understand the connection.

In this study, we aimed to identify and validate the differentially expressed circular RNAs in MDD patients, assess their utility as biomarkers, and investigate the possible processes of circRNAs in MDD.

## Materials and Methods

### Subjects

We recruited 29 MDD patients and 19 healthy controls from June to September 2020. MDD patients were recruited through the Department of Psychology at the Affiliated Hospital of Guangdong Medical University. Participants or their legal guardians were informed of the trial’s benefits and risks before providing written informed permission. All MDD patients met the following inclusion criteria: (1) met the MDD diagnostic criteria of *Manual of Mental Disorders (5th Edition) (DSM-V)*; (2) the 24-item Hamilton Depression Scale (HAMD-24) score ≥ 8, Self-rating depression scale (SDS) score ≥ 53; (3) age 14 to 59 years, and the age of MDD onset under 55 years; (4) Han people; (5) depression patients without any medications or not taking any psychiatric medication for at least three months; (6) no infectious history, no significant physical disorder; (7) no history of smoking or alcohol abuse; (8) no history of head trauma. And the following exclusion criteria were applied for the MDD patients: (1) with other mental disorders, e.g., substance abuse, schizophrenia, obsessive-compulsive disorder, etc.; (2) pregnant or lactating female; (3) long-term use of drugs or health care products. The healthy controls were recruited in the community and the inclusion criteria were as follows: (1) the 24-item Hamilton Depression Scale (HAMD-24) score < 8, Self-rating depression scale (SDS) score < 53; (2) age 18 to 59 years; (3) Han people; (4) not taking any medication for at least three months; (5) no history of mental disorder and no family history of major mental disorder, no significant physical disorder; (6) no history of smoking or alcohol abuse. The following exclusion criteria were applied for the healthy controls: (1) pregnant or lactating female; (2) long-term use of drugs or health care products. All participants were jointly diagnosed by two or more psychiatrists. The demographic data of the patients and controls are shown in Table 1. The differences between MDD patients and healthy controls were not significant in age, gender ratio, or ethnicity, but the HAMD scores were significantly different.

**TABLE 1:** Clinical characteristics of MDD patients and healthy controls.

Variable	HC(*n* = 19)	MDD(*n* = 29)	*p*-value	Statistical analyses

Ethnicity	Han	Han		
Age (years)	22.74 (2.47)	22.1 (5.27)	0.586	Unpaired *t*-test with Welch’s correction
Female	13 (68.42%)	21(72.41%)	> 0.9999	Fisher’s exact test
HAMD-17 score	0.7778 (1.84)	16.52 (4.71)	< 0.0001	Mann whitney test
HAMD-24 score	0.9444 (1.87)	21.17 (6.84)	< 0.0001	Mann whitney test
HAMA	0.8889 (1.33)	16.66 (6.14)	< 0.0001	Mann whitney test

*Data presented as mean (standard deviation) or number of participants in each group (% of total). MDD, major depressive disorder; HC, healthy control; HAMD-17, the 17-item Hamilton Depression Scale; HAMD-24, the 24-item Hamilton Depression Scale; HAMA, the Hamilton Anxiety Scale.*

### Whole Transcriptome Sequencing

Following an overnight fast, venous blood was taken from each participant. EDTA anticoagulant tubes were used to collect whole blood samples, and whole transcriptome sequencing was done on blood samples from 4 MDD patients and 4 healthy controls. CircRNAs exhibiting | log2(fold change) | > 1 and p-value < 0.05 were identified as significant. Use the FPKM value to identify the mRNA of each coding gene. Differentially expressed mRNAs were identified based on | log2(fold change) | > 0.58 with a *p*-value < 0.05, and at least one sample of the gene FPKM value ≥ 1.

### RNA Extraction and Real-Time Quantitative Reverse Transcription PCR

Total RNA was extracted by Trizol (Invitrogen, United States). According to the manufacturer’s protocol affiliated with Evo M-MLV RT Kit with gDNA Clean for qPCR II (Accurate Biology, China), circRNA was reverse transcribed to complementary cDNA and then quantified by SYBR Green Real-time PCR Master Mix (Accurate Biology, China). β-actin was used as the endogenous control, detailed information about these primers was provided in Supplementary Table 1. The expression levels of circRNAs were normalized to β-actin, and the relative expression levels of circRNAs were shown by the value of the 2^–ΔΔCt^.

### Enzyme-Linked Immunosorbent Assay (ELISA) Analyses

The plasma was extracted by centrifugation at 3,000 rpm, 27°C for 10 min. By the manufacturer’s protocols, ELISA kits (Multi Sciences, China) were used to detect the plasma concentration of BDNF, GDNF, and β-NGF. The maximum absorption wavelength at 450 nm and the reference wavelength at 570 nm was measured using a microplate reader (BioTek Epoch, United States), then subtracting readings at 570 nm from the readings at 450 nm as calibration optical density (OD) value.

### Bioinformatics Analysis

Based on miRanda^1^, we predicted potential miRNA targets for the target circRNAs. The potential mRNA targets of the miRNAs were predicted by TargetScan^2^). Cytoscape^3^ was used to delineate the circRNA–miRNA–mRNA network. Using clusterProfiler package to perform gene ontology (GO) analysis to analyze the potential function of the target gene. Kyoko Encyclopedia of Genes and Genomes (KEGG) enrichment analysis analyzed the related pathways of target genes through KOBAS 3.0^4^.

### Statistical Analysis

The difference of candidate circRNAs expression between MDD patients and healthy controls was evaluated by t-test. Demographic variables were compared between MDD patients and healthy controls with unpaired *t*-test with Welch’s correction, Fisher’s exact test, or Mann Whitney test. All tests were two-sided and P-value < 0.05 was considered statistically significant. Statistical analysis was performed with GraphPad Prism (GraphPad Software, United States) version 8.42.

## Results

### CircRNAs Expression Profile in Major Depressive Disorder Patients and Healthy Controls

**FIGURE 1:**
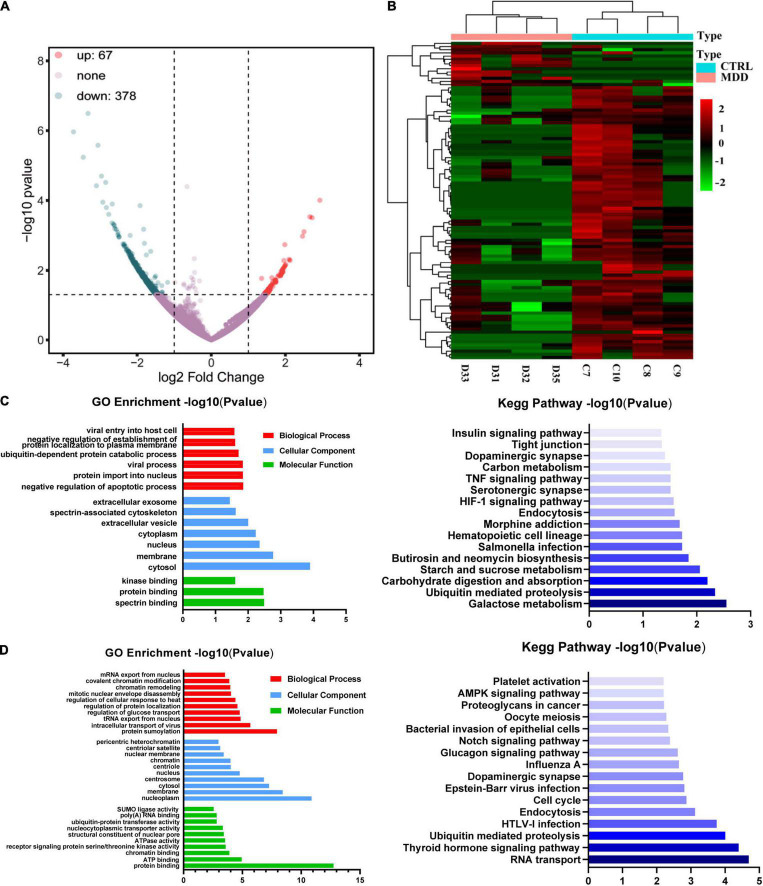
CircRNA expression profiles. **(A)** Volcano plot of dysregulated circRNAs between 4 MDD patients and 4 healthy controls. **(B)** Heatmap of top 100 dysregulated circRNAs between 4 MDD patients and 4 healthy. **(C)** GO and KEGG enrichment analyses based on upregulated circRNAs in MDD patients. **(D)** GO and KEGG enrichment analyses based on downregulated circRNAs in MDD patients.

We used whole transcriptome sequencing to analyze blood samples from four MDD patients and four healthy controls and identified 445 circRNAs expressing differentially (378 downregulated and 67 upregulated) (Figure 1A). The expression profiles of circRNAs in MDD patients and healthy controls exhibited good consistency, as shown in the heat map analysis of the top 100-dysregulated circRNAs (Figure 1B).

### Enrichment Analyses of Dysregulated CircRNAs

Gene ontology enrichment analysis and KEGG enrichment analysis based on upregulated circRNAs were shown in Figure 1C. In the GO enrichment results of upregulated circRNAs, negative regulation of apoptotic process (GO: 0043066), protein import into nucleus (GO: 0006606), viral process (GO: 0016032), ubiquitin-dependent protein catabolic process (GO: 0006511), negative regulation of establishment of protein localization to plasma membrane (GO: 0090005) and viral entry into host cell (GO: 0046718) were the mainly enriched biological process terms. In addition, KEGG enrichment analysis (Figure 1C) showed that upregulated circRNAs were enriched in neuron-related pathways: serotonergic synapses (hsa04726) and dopaminergic synapses (hsa04728). GO enrichment analysis and KEGG enrichment analysis based on downregulated circRNAs were shown in Figure 1D. The top five biological process terms of downregulated circRNAs were as follows: protein sumoylation (GO: 0016925), intracellular transport of virus (GO: 0075733), tRNA export from nucleus (GO: 0006409), regulation of glucose transport (GO: 0010827) and regulation of protein localization (GO: 0032880). The first 5 pathways of KEGG enrichment analysis showed that downregulated circRNAs were enriched in RNA transport (hsa03013), thyroid hormone signaling pathway (hsa04919), ubiquitin mediated proteolysis (hsa04120), HTLV-I infection (hsa05166), and endocytosis (hsa04144).

### Validation of 20 Dysregulated Candidate CircRNAs

**FIGURE 2:**
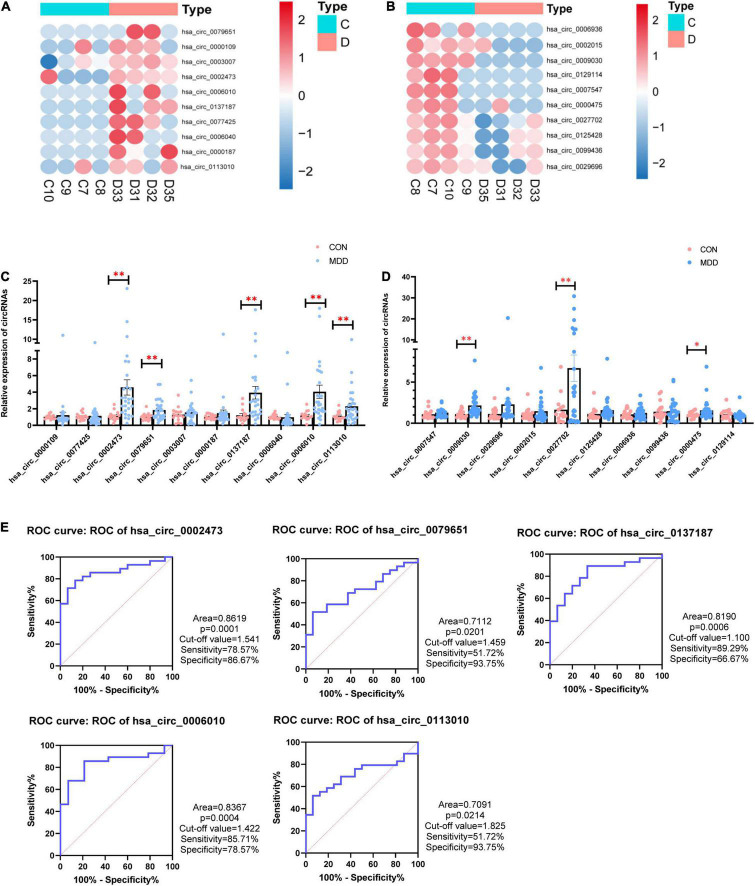
20 candidate CircRNAs (top 10 upregulated and top 10 downregulated) in MDD patients and healthy controls. **(A)** Heatmaps of top 10 upregulated circRNAs. **(B)** Heatmaps of top 10 downregulated circRNAs. **(C)** Comparison of top 10 upregulated circRNAs relative expressions between 29 MDD patients and 19 healthy controls. **(D)** Comparison of top 10 downregulated circRNAs relative expressions between 29 MDD patients and 19 healthy controls. **(E)** ROC curve analyses of 5 verified candidate circRNAs for predicting MDD risk. Data are presented as mean ± standard error of the mean (SEM). *p < 0.05, **p < 0.01.

We selected 20 dysregulated circRNAs (the top 10 upregulated and the top 10 downregulated) for further study (Table 2). Heat map analysis of the 10 upregulated circRNAs (Figure 2A) and the 10 downregulated circRNAs (Figure 2B) showed that both could distinguish MDD patients from healthy controls. The 20 dysregulated circular RNAs were further verified by RT-qPCR in 29 MDD patients and 19 healthy controls, subsequently, 5 upregulated circRNAs were consistent with circRNAs sequencing among the 20 dysregulated circRNAs: hsa_circ_0002473, hsa_circ_0079651, hsa_circ_0137187, hsa_circ_0006010 and hsa_circ_0113010 (Figures 2C,D). The receiver operating characteristic curve (ROC) analysis was performed on these five circRNAs to assess their diagnostic capability for MDD (Figure 2E). All of them were able to discriminate between MDD patients and healthy controls, and hsa_circ_0002473 and hsa_circ_0006010 exhibited better predictive capacity of biomarkers (AUC = 0.8619 and AUC = 0.8367, respectively).

**TABLE 2:** Twenty dysregulated circRNAs in whole transcriptome sequencing were selected.

CircRNA_id	CircBase_id	hg38_positon	Gene	log2Fold -Change	*p*-value	padj	Trend
chr1:112653597-112659779 +	hsa_circ_0000109	chr1:113196219-113202401+	CAPZA1	2.661389358	0.000294984	0.469082864	Up
chr6:99439768-99465136-	hsa_circ_0077425	chr6:99887644-99913012-	USP45	2.723704441	0.000312774	0.469082864	Up
chr13:95723241-95725252 +	hsa_circ_0002473	chr13:96375495-96377506+	DNAJC3	2.504480477	0.00078066	0.685321985	Up
chr7:26195846-26197732-	hsa_circ_0079651	chr7:26235466-26237352-	HNRNPA2B1	2.46293726	0.001049879	0.685321985	Up
chr15:100564691-100565265-	hsa_circ_0003007	chr15:101104896-101105470-	LINS1	1.959125317	0.005230603	0.685321985	Up
chr1:222823883-222835215 +	hsa_circ_0000187	chr1:222997225-223008557+	AL392172.1	2.01109853	0.006768998	0.685321985	Up
chr8:81713917-81718224-	hsa_circ_0137187	chr8:82626152-82630459-	ZFAND1	1.991446071	0.007293083	0.685321985	Up
chr3:197830769-197839397 +	hsa_circ_0006040	chr3:197557640-197566268+	LRCH3	1.96759118	0.007978386	0.685321985	Up
chr7:32632542-32639365-	hsa_circ_0006010	chr7:32672154-32678977-	DPY19L1P1	1.94697564	0.008616892	0.685321985	Up
chr1:29109335-29115803 +	hsa_circ_0113010	chr1:29435847-29442315+	EPB41	1.938721876	0.011028077	0.685321985	Up
chr13:21161789-21172681-	hsa_circ_0029696	chr13:21735928-21746820-	SKA3	−3.32816757	0.000000320	0.007690326	down
chr13:21157921-21172681-	hsa_circ_0007547	chr13:21732060-21746820-	SKA3	−3.72104263	0.000001083	0.012990455	down
chr12:89466769-89472275-	hsa_circ_0027702	chr12:89860546-89866052-	POC1B	−3.059771756	0.000002630	0.021036112	down
chr5:131698916-131709272-	hsa_circ_0009030	chr5:131034609-131044965-	AC008695.1	−3.456438458	0.000005791	0.034740473	down
chr4:143527867-143530526 +	hsa_circ_0125428	chr4:144449020-144451679+	SMARCA5	−2.965370872	0.000020046	0.096203806	down
chr12:89459637-89472275-	hsa_circ_0099436	chr12:89853414-89866052-	POC1B	−2.851444921	0.000030462	0.120266704	down
chr17:55401468-55403868-	hsa_circ_0002015	chr17:53478829-53481229-	MMD	−3.102035597	0.000037882	0.120266704	down
chr6:149771161-149773169 +	hsa_circ_0006936	chr6:150092297-150094305+	PCMT1	−2.928561379	0.000112222	0.269287152	down
chr5:53646232-53658624 +	hsa_circ_0129114	chr5:52942062-52954454+	NDUFS4	−2.769203899	0.000250523	0.429395568	down
chr13:37040404-37051583-	hsa_circ_0000475	chr13:37614541-37625720-	SUPT20H	−2.833288838	0.000160359	0.295997981	down

*CircBase_id, the id of circRNAs in circBase database; hg38_positon, the position of circRNAs in Genome Reference Consortium Human Build 38; padj, adjusted p-value.*

### Functional Prediction of the ceRNA Network of Verified Candidate CircRNAs

**FIGURE 3:**
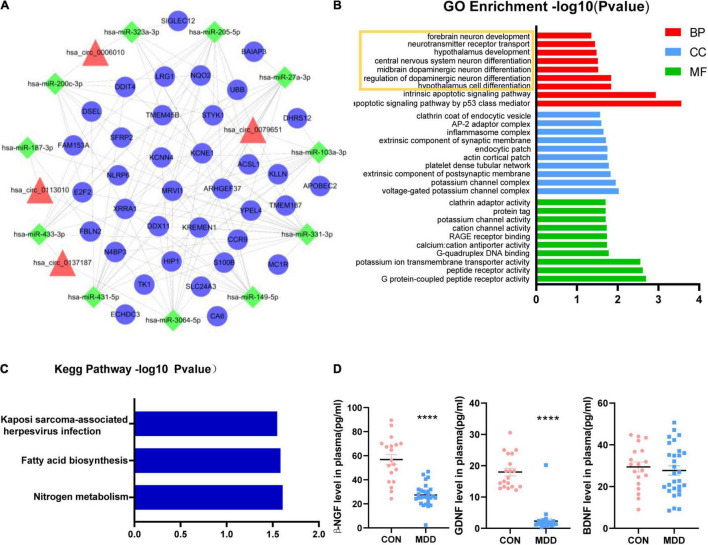
Functional prediction of circHNRNPA2B1, circZFAND1, circDPY19L1P1, and circEPB41. **(A)**: CircRNA-miRNA-mRNA network. Four candidate circRNAs with their potential target 11 miRNAs and 36 mRNAs. Triangles represent circRNAs, squares represent miRNAs and circles represent mRNAs. **(B)** Part of GO enrichment analysis items based on 36 mRNAs that related to ceRNA network. **(C)** KEGG enrichment analysis based on 36 mRNAs that related to ceRNA network. **(D)** The plasma concentration of β-NGF, GDNF, and BDNF between 29 MDD patients and 19 healthy controls. Data are presented as mean ± standard error of the mean (SEM). ****p < 0.0001.

All the sequence information of human miRNA was downloaded from the miRBase database^5^, and then miRanda software was used to predict the binding relationship between circRNAs and miRNAs. The binding relationship between miRNAs and mRNAs was predicted using the binding relationship between miRNA and mRNA in the TargetScan database. For the mRNAs we used 150 genes that had been detected to be upregulated in whole transcriptome sequencing (Supplementary Table 2). Based on these five circRNAs, we obtained a ceRNA network, including 11 miRNA and 36 mRNA (Figure 3A). Among them, we found hsa_circ_0002473 had no ceRNA relationship pair. The probable roles of these 36 mRNAs connected to the ceRNA network were predicted using GO and KEGG enrichment analysis. These mRNAs were linked to the growth and differentiation of neurons, according to biological process items (Figure 3B): hypothalamus cell differentiation (GO: 0021979), regulation of dopaminergic neuron differentiation (GO: 1904338), midbrain dopaminergic neuron differentiation (GO: 1904948), central nervous system neuron differentiation (GO: 0021953), hypothalamus development (GO: 0021854), neurotransmitter receptor transport (GO: 0099637) and forebrain neuron development (GO: 0021884). In terms of cellular components, these mRNAs were enriched in voltage-gated potassium channel complex (GO: 0008076), potassium channel complex (GO: 0034705), extrinsic component of postsynaptic membrane (GO: 0098890), and so on. In addition, these mRNAs were enriched in molecular function such as G protein-coupled peptide receptor activity (GO: 0008528), peptide receptor activity (GO: 0001653), potassium ion transmembrane transporter activity (GO: 0015079), and G-quadruplex DNA binding (GO: 0051880), etc. Regarding KEGG pathways based on target mRNAs (Figure 3C), it was disclosed that 36 mRNAs were enriched in nitrogen metabolism (hsa00910) and fatty acid biosynthesis (hsa00061).

### Neurotrophic Factors β-NGF, GDNF Were Decreased in the Plasma of Major Depressive Disorder Patients

The volume and density of neurons in the dorsolateral prefrontal cortex (dlPFC) and dentate gyrus (DG) were observed to be lower in MDD patients (Rajkowska, 2000; Boldrini et al., 2013) which was consistent with our KEGG enrichment analysis (Figure 1C) and biological process prediction (Figure 3B). Neurotrophic factors (NF) such as Glial cell-derived neurotrophic factor (GDNF), beta-nerve growth factor (β-NGF), and brain-derived neurotrophic factor (BDNF), play key factors in promoting neuron growth, maturing, and repairing (Allen et al., 2013; Xiao and Le, 2016) and MDD patients have neurotrophic factor disorders (Shi et al., 2020). We wondered if there are similar changes in the samples we collected. Therefore, we indirectly evaluate the changes in brain neurons by detecting the plasma neurotrophic factors. The results of plasma ELISA (Figure 3D) showed that β-NGF, GDNF was decreased in MDD patients and BDNF was unchanged in MDD patients. In addition, we found that the concentration of β-NGF and GDNF were not associated with the relative expression of these 5 circRNAs.

## Discussion

In this study, we performed whole transcriptome sequencing on the whole blood RNA of MDD patients and healthy controls: 67 upregulated circRNAs and 378 downregulated circRNAs (Figure 1A). In addition, upregulated circRNAs were enriched in neuron-related pathways: serotonergic synapses and dopaminergic synapses (Figure 1C). Downregulated circRNAs were enriched in RNA transport, thyroid hormone signaling pathway, ubiquitin mediated proteolysis, HTLV-I infection, and so on (Figure 1D). Then, we selected 20 dysregulated circRNAs (the top 10 upregulated and the top 10 downregulated) (Table 2) for further study. Five upregulated circRNAs: hsa_circ_0002473, hsa_circ_0079651, hsa_circ_0137187, hsa_circ_0006010, and hsa_circ_0113010 were specifically expressed in MDD patients (Figure 2C), and could distinguish MDD patients from healthy controls (Figure 2E). We did not validate circRNAs with the same expression trend in the down-regulated circRNAs (Figure 2D). According to the present research, hsa_circ_0002473, hsa_circ_0079651, hsa_circ_0137187, hsa_circ_0006010, and hsa_circ_0113010 might be novel biomarkers for MDD.

CircRNAs can act as miRNA sponges, preventing miRNAs from binding and suppressing target mRNA (Chen, 2020). In this study, these five circRNAs were discovered for the first time. For these five circRNAs, we constructed a ceRNA network, and this ceRNA network revealed that 36 mRNA may reflect the function of these circRNAs in MDD (Figure 3A). We discovered that hsa_circ_0002473 has no ceRNA relationship, suggesting it does not function as a ceRNA. Biological process items showed that these 36 mRNAs were related to the growth and differentiation of neurons (Figure 3B): regulation of dopaminergic neuron differentiation, midbrain dopaminergic neuron differentiation, central nervous system neuron differentiation, hypothalamus development, neurotransmitter receptor transport, and forebrain neuron development, which was consistent with the KEGG enrichment analysis of the total upregulated circRNAs (Figure 1C). It implies that there may be neuronal changes in MDD patients. Moreover, the size and density of the dorsolateral prefrontal cortex (dlPFC) and dentate gyrus (DG) neurons in MDD patients were reduced (Rajkowska, 2000; Boldrini et al., 2013). There was evidence that neurotrophic factors regulate neurogenesis, and MDD patients often present with neurotrophic factor disorders (Shi et al., 2020). We would like to find out whether neurotrophic factors are affected in this study and if circRNAs are related to neurotrophic factors.

It is known that neurotrophic factors are able to promote the growth, proliferation, differentiation, and survival of neurons; neurotrophic factors such as BDNF, GDNF, and NGF are dysregulated in MDD patients and are important in antidepressant drug mechanisms (Saavedra et al., 2008; Allen et al., 2013; Song et al., 2017; Sun et al., 2019; Castrén and Monteggia, 2021). In this study, β-NGF and GDNF levels decreased in MDD patients, while BDNF levels were unaffected (Figure 3D). We also found that β-NGF and GDNF concentrations do not affect the relative expression of these five circRNAs. There is still no consensus on how these three neurotrophic factors differ in MDD individuals. The majority of research showed that BDNF expression in MDD decreased (de Azevedo Cardoso et al., 2014; Phillips, 2017; Kojima et al., 2019; Teng et al., 2021). The results of our experiment contradicted this. This could be due to different populations in our experiment, or it could be due to a lack of volunteers, resulting in a false negative. Regarding GDNF, one study found the level of GDNF was decreased in young MDD patients (age 13–24 years) but not in old MDD patients (age 25–45 years) (Sun et al., 2019). According to two meta-analyses, NGF levels were significantly lower in MDD than in healthy controls (Chen et al., 2015; Wiener et al., 2015; Çakici et al., 2020), which was consistent with our finding. Another study of depression in adolescent patients discovered increased BDNF but no changes in NGF or GDNF (Bilgic et al., 2020). Taken together, these findings indicate the existence of aberrant neurotrophic factors in patients with MDD, which may be a significant etiology of major depression or simply a “consequence” of the stress response to depression, and restoring neurotrophic factor homeostasis may help relieve depression symptoms.

The above information suggested that hsa_circ_0079651, hsa_circ_0137187, hsa_circ_0006010, and hsa_circ_0113010 are suspected of being involved in the neuroplasticity of MDD, although the specific mechanism requires additional investigation. In the ceRNA network, 11 related miRNAs were discovered (Figure 3A); among them, hsa-miR-103a-3p (Devoto et al., 2020), hsa-miR-27a-3p (Sala Frigerio et al., 2013), and hsa-miR-200c-3p (Lee et al., 2018) were abnormal in neuropsychiatric diseases in previous investigations. Among the 36 mRNAs associated with the ceRNA network (Figure 3A), 3 mRNAs had been reported to be related to depression (DDIT4, S100B, Fbln2). DDIT4 levels were elevated in MDD patients and had been verified to be related to neuronal atrophy and depressive behavior caused by chronic stress (Malagelada et al., 2011; Ota et al., 2014; Wang et al., 2018; Perez-Sisques et al., 2021); S100B levels were also elevated in MDD patients, and associated with glial cell dysregulation (Gos et al., 2013; Güleş et al., 2020; O’Leary and Mechawar, 2021; Zhang et al., 2021). Reduced Fbln2 alleviates the state of cognitive impairment and neural function in mice with chronic unpredictable mild stress (CUMS) through inhibiting the TGF-β1 signaling pathway (Tang et al., 2018). Additionally, 15 mRNAs had been linked to neurogenesis or neuropsychiatric disorders (Table 3). These investigations back up the current findings that hsa_circ_0079651, hsa_circ_0137187, hsa_circ_0006010, and hsa_circ_0113010 are linked to neuroplasticity in MDD. However, just a few studies have delved into the mechanisms of circRNAs in depression. One study found circDYM improved depressive-like behavior in mice through inhibition of miR-9 activity and leading to increased expression of the downstream target HECTD1 and increase of HSP90 ubiquitination then reducing microglia activation (Zhang et al., 2018). The gut microbiota of NLRP3 KO mice transplanted into chronic unpredictable stress (CUS) mice modulated astrocyte dysfunction and improved depression-like behavior in CUS mice *via* circHIPK2 (Zhang et al., 2019). CircSTAG1 was also found to alleviate depressive-like behavior in CUS mice: circSTAG1 trapped ALKBH5 and reduced its translocation to the nucleus, leading to increased m6A methylation of FAAH mRNA and degradation of FAAH in astrocytes, followed by alleviation of depressive-like behavior in mice (Huang et al., 2020). Likewise, our study provides clues to the role of circular RNAs in MDD: circRNAs may affect neuroplasticity through ceRNA mechanisms in MDD patients, and their specific pathway in MDD merit more study.

**TABLE 3:** The potential function of 15 mRNAs in neurogenesis or neuropsychiatric disorders based on previous studies.

mRNA	Study ID	Potential function
BAIAP3	Wojcik et al., 2013	BAIAP3 was associated with anxiety and changes in response to benzodiazepines.
CCR9	Liu et al., 2007; Cao et al., 2012	CCR9 had neuroprotective effects on mouse hippocampal neurons.
E2F2	Castillo et al., 2015; Wu et al., 2015	E2F2 was related to nerve repair after spinal cord injury and repairing neuronal cell DNA damage.
ECHDC3	Tan et al., 2021	ECHDC3 was related to cranial nerve degeneration.
HIP1	Peng et al., 2017	HIP1R was involved in the development of neuronal dendrites and the formation of excitatory synapses.
KCNE1	Jamali et al., 2006; Hussain et al., 2010	KCNE1 was reduced in the entorhinal cortex (EC) of mesial temporal lobe epilepsies (MTLE) patients; the interaction of KCNE1 and Rap2 plays a key role in maintaining the morphological integrity of neuronal dendrites and synaptic transmission.
KCNN4	Skaper, 2011; Sugunan et al., 2016	KCNN4 channel may be a drug target in neurological diseases.
KREMEN1	Wu and Murashov, 2013; Wang et al., 2019	Decreasing the expression of kremen1 had a protective effect on neurons.
LRG1	Miyajima et al., 2013; Akiba et al., 2017	Increased concentration of LRG in cerebrospinal fluid is related to the decline of human cognitive ability; overexpression of hippocampal LRG can lead to synaptic dysfunction and memory impairment.
MC1R	Catania, 2008; Chen et al., 2017	MC1R had a protective effect on neurons and the nigrostriatal dopaminergic system.
N4BP3	Schmeisser et al., 2013; Kiem et al., 2017	N4BP3 had important functions in the development of neurites.
NLRP6	Li et al., 2019; Zhang et al., 2020	NLRP6 regulated the survival of neurons.
SFRP2	Aubert et al., 2002; Amura et al., 2005; Kele et al., 2012	SFRP2 regulated the development of neurons and embryonic stem cells.
SLC24A3	Zhou et al., 2011	SLC24A3 was highly specific in the substantia nigra of the adult rat brain.
UBB	Jung et al., 2018; Park et al., 2020	Ubb is related to the differentiation of neural stem cell (NSC)

To sum up the above, we found the disorder of circRNAs in the blood of MDD patients and preliminarily demonstrated that hsa_circ_0002473, hsa_circ_0079651, hsa_circ_0137187, hsa_circ_0006010, and hsa_circ_0113010 were highly expressed in MDD patients and could be used as diagnostic markers for MDD. Among them, hsa_circ_0079651, hsa_circ_0137187, hsa_circ_0006010, and hsa_circ_0113010 that may affect the neuroplasticity of MDD through the ceRNA mechanism, and their exact pathway in MDD deserve further study.

## Data Availability Statement

The datasets presented in this study can be found in online repositories. The names of the repository/repositories and accession number(s) can be found below: https://www.ncbi.nlm.nih.gov/geo/query/acc.cgi?acc=GSE190518.

## Ethics Statement

The studies involving human participants were reviewed and approved by Ethics Committee of Guangdong Medical University. Written informed consent to participate in this study was provided by the participants’ legal guardian/next of kin.

## Author Contributions

YC and LC designed the study and conceptualization. YC, LC, DZ, and YJ finished the original draft. ZL, DZ, YW, QP, JL, JY, and HL diagnosed and collected samples. DZ, YJ, RC, and XC did the investigation and statistical analysis. YC and LC revised the investigation and methodology. All authors contributed to the article and approved the submitted version.

## Conflict of Interest

The authors declare that the research was conducted in the absence of any commercial or financial relationships that could be construed as a potential conflict of interest.

## Publisher’s Note

All claims expressed in this article are solely those of the authors and do not necessarily represent those of their affiliated organizations, or those of the publisher, the editors and the reviewers. Any product that may be evaluated in this article, or claim that may be made by its manufacturer, is not guaranteed or endorsed by the publisher.

## Footnotes

1
http://www.microrna.org/microrna/home.do

2
http://www.targetscan.org/

3
http://cytoscape.github.io/

4
http://kobas.cbi.pku.edu.cn/

5
https://mirbase.org/
