# Supplementary material for: Peripheral Blood Circular RNAs as a Biomarker for Major Depressive Disorder and Prediction of Possible Pathways
Source: Front Neurosci. 2022 Mar 31;16:844422. doi: 10.3389/fnins.2022.844422 (PMC9009243; doi:10.3389/fnins.2022.844422)
Supplement: Supplementary file 1 [file Table_1.docx]

Supplementary Material

# Supplementary Tables

Supplementary Table S1. List of primers used for RT-qPCR.

| **gene** | **circBase_id** | **Forward primer** | **Reverse primer** |
| --- | --- | --- | --- |
| USP45 | hsa_circ_0077425 | ACTTGTAAGATCTCCACGGTGA | TGGCAGCTCTAGGTTGATGA |
| CAPZA1 | hsa_circ_0000109 | TGCTTAAGATTCAGTGCATTTG | GGGTCACTTGCTTCTTTCCG |
| DNAJC3 | hsa_circ_0002473 | AAAGTGATGGTGACCCTGATAAC | GCAGCTTTTGATTTGCCCAT |
| HNRNPA2B1 | hsa_circ_0079651 | TTTGGCTTTGGGGATTCACG | TTTTCTCTCTCCAGGTCCTCC |
| AL392172.1（DISP1） | hsa_circ_0000187 | TAAAAGGAGTTGAGAAGAGGAGG | TCAAGTATTCCCAGTAACAGTGT |
| ZFAND1 | hsa_circ_0137187 | ACCACAGATTTTCTTCCATTTGT | GAGACTCCCTGCTTCTGTGT |
| LRCH3 | hsa_circ_0006040 | ACATTTGCCTGAAGACCTGTC | CACAAGTGTACCGGCAATGT |
| LINS1 | hsa_circ_0003007 | CAACAAGACACATGCGGACA | CTCCATGTCTTTGAGCTTCCA |
| DPY19L1P1 | hsa_circ_0006010 | TTGCAGTGTATGTTGTCGGG | TCCACATTACACAGGTACACATG |
| EPB41 | hsa_circ_0113010 | AGCTCAGACTGACGACAACA | TTTGAGTTGTGGTGGTGCTG |
| SKA3 | hsa_circ_0007547 | CCAGAAGATATTCTCCAGACTTTGA | TGTTGCCTTTATGAAATCAATGCC |
| AC008695.1（FNIP1） | hsa_circ_0009030 | ATTCAGTTCACAGCAACCCA | GTGAGGAGTTTGGGGAAGGA |
| SKA3 | hsa_circ_0029696 | CCCCATCATCCAGCAGTTG | TCTCATTGGATAATCTTCAAAGTCACT |
| MMD | hsa_circ_0002015 | GGTGACATCAATGGTTAAATCT | AGCCAGATAAACCAACGCATAT |
| POC1B | hsa_circ_0027702 | ACCCAAGAACACCACATCCC | TCGGGTGAAAATTTCTACAGTCT |
| SMARCA5 | hsa_circ_0125428 | ACACCTCTTCAGAACAACTTGC | AAAGCAGCCATATGAAGCCTC |
| PCMT1 | hsa_circ_0006936 | CCACAATCAATAGGTTTCCAAGC | TGATTCCATTTTCATGTGTGG |
| POC1B | hsa_circ_0099436 | ACCCAAGAACACCACATCCC | CCATCGGGTGAAAATTGTGGT |
| SUPT20H（FAM48A） | hsa_circ_0000475 | CACAAATGGACCCAGTACTGT | GCTGATGAAGTGGGGTGAAC |
| NDUFS4 | hsa_circ_0129114 | CCTTTGATGGGTTGGGCATC | ACTCCAGTTAAAGTAGTGATATCCAT |
| Human β-actin | | CATGTACGTTGCTATCCAGGC | CTCCTTAATGTCACGCACGAT |

**Supplementary Table S2**. In whole transcriptome sequencing, 150 genes were shown to be elevated in 4 MDD patients compared to healthy controls. Ensembl_id, the id of mRNAs in Ensembl database; gene_name, gene symbol standard name; padj, adjusted p-value.

| **ensembl_id** | **gene_name** | **log2FoldChange** | **p-value** | **padj** |
| --- | --- | --- | --- | --- |
| ENSG00000185710 | SMG1P4 | 2.917749985 | 4.90E-29 | 1.59123E-24 |
| ENSG00000166435 | XRRA1 | 1.797456708 | 1.07376E-16 | 1.74454E-12 |
| ENSG00000278879 | AP000560.1 | 1.652036627 | 1.58123E-05 | 0.090214305 |
| ENSG00000173295 | FAM86B3P | 0.609495349 | 0.000535974 | 0.844688418 |
| ENSG00000226862 | AC104463.2 | 1.270389301 | 0.000545899 | 0.844688418 |
| ENSG00000131686 | CA6 | 1.927415779 | 0.000720694 | 0.936729707 |
| ENSG00000238260 | AL513320.1 | 1.220167829 | 0.000965019 | 0.955736826 |
| ENSG00000231970 | AL355490.2 | 1.641903168 | 0.001714242 | 0.999859665 |
| ENSG00000283378 | CNTNAP3C | 1.592625526 | 0.001915429 | 0.999859665 |
| ENSG00000271335 | AL117336.3 | 0.627600807 | 0.001946828 | 0.999859665 |
| ENSG00000255760 | LINC02422 | 0.830197614 | 0.001952925 | 0.999859665 |
| ENSG00000226266 | AC009961.1 | 0.699787093 | 0.002097164 | 0.999859665 |
| ENSG00000211938 | IGHV3-7 | 0.755978796 | 0.00211726 | 0.999859665 |
| ENSG00000259033 | AL356804.1 | 3.904337479 | 0.002136458 | 0.999859665 |
| ENSG00000264754 | AC110921.1 | 0.913606788 | 0.002348478 | 0.999859665 |
| ENSG00000227945 | AL590006.1 | 1.344560429 | 0.002606308 | 0.999859665 |
| ENSG00000233038 | AC011899.2 | 0.796221332 | 0.002851045 | 0.999859665 |
| ENSG00000124701 | APOBEC2 | 1.417997812 | 0.003148417 | 0.999859665 |
| ENSG00000280198 | AC087163.3 | 1.170188679 | 0.003578733 | 0.999859665 |
| ENSG00000267984 | AC008750.4 | 1.04548641 | 0.003718055 | 0.999859665 |
| ENSG00000196502 | SULT1A1 | 1.233734505 | 0.004013947 | 0.999859665 |
| ENSG00000100290 | BIK | 1.14792324 | 0.004018872 | 0.999859665 |
| ENSG00000233429 | HOTAIRM1 | 1.141518084 | 0.004341158 | 0.999859665 |
| ENSG00000163520 | FBLN2 | 0.978796561 | 0.004919883 | 0.999859665 |
| ENSG00000102796 | DHRS12 | 0.599345941 | 0.005199617 | 0.999859665 |
| ENSG00000273363 | AL353801.3 | 0.818920393 | 0.005204578 | 0.999859665 |
| ENSG00000251095 | AC097478.1 | 0.770490702 | 0.005312977 | 0.999859665 |
| ENSG00000013583 | HEBP1 | 0.923513262 | 0.005417737 | 0.999859665 |
| ENSG00000240666 | MME-AS1 | 1.369581825 | 0.005556801 | 0.999859665 |
| ENSG00000263307 | AC007216.4 | 0.970164973 | 0.005936214 | 0.999859665 |
| ENSG00000281106 | TMEM272 | 0.680909571 | 0.006344551 | 0.999859665 |
| ENSG00000281376 | ABALON | 0.716295489 | 0.00635209 | 0.999859665 |
| ENSG00000104783 | KCNN4 | 0.724881258 | 0.006401932 | 0.999859665 |
| ENSG00000267364 | AC022706.1 | 0.80001835 | 0.006627892 | 0.999859665 |
| ENSG00000213741 | RPS29 | 0.93854829 | 0.006813089 | 0.999859665 |
| ENSG00000174885 | NLRP6 | 0.970790485 | 0.008086945 | 0.999859665 |
| ENSG00000229930 | AC138393.2 | 4.638447964 | 0.008442906 | 0.999859665 |
| ENSG00000170074 | FAM153A | 0.672332434 | 0.008647893 | 0.999859665 |
| ENSG00000230257 | NFE4 | 0.978875868 | 0.008837157 | 0.999859665 |
| ENSG00000107833 | NPM3 | 0.678360287 | 0.008864098 | 0.999859665 |
| ENSG00000257210 | NACAP8 | 2.979604843 | 0.008897657 | 0.999859665 |
| ENSG00000261288 | AC093525.5 | 1.577121143 | 0.008929843 | 0.999859665 |
| ENSG00000251139 | AC084871.1 | 1.387077858 | 0.00901978 | 0.999859665 |
| ENSG00000211795 | TRAV8-6 | 0.79692463 | 0.009420526 | 0.999859665 |
| ENSG00000269427 | AC024075.3 | 1.620679685 | 0.009537779 | 0.999859665 |
| ENSG00000183111 | ARHGEF37 | 0.70021576 | 0.009768921 | 0.999859665 |
| ENSG00000246273 | SBF2-AS1 | 0.609705627 | 0.0099033 | 0.999859665 |
| ENSG00000258745 | AL358334.3 | 1.177659713 | 0.01049679 | 0.999859665 |
| ENSG00000215196 | BASP1-AS1 | 1.517554905 | 0.010641955 | 0.999859665 |
| ENSG00000224067 | AL354877.1 | 1.402586699 | 0.010721911 | 0.999859665 |
| ENSG00000275927 | AC009152.1 | 0.688099616 | 0.0113148 | 0.999859665 |
| ENSG00000259845 | HERC2P10 | 3.129956082 | 0.011604241 | 0.999859665 |
| ENSG00000240793 | UBA52P8 | 2.276184434 | 0.011982724 | 0.999859665 |
| ENSG00000268615 | AL353803.5 | 1.908066882 | 0.012028001 | 0.999859665 |
| ENSG00000227268 | KLLN | 0.681201225 | 0.01277057 | 0.999859665 |
| ENSG00000237803 | LINC00211 | 0.713582118 | 0.01292139 | 0.999859665 |
| ENSG00000183762 | KREMEN1 | 1.418021682 | 0.014218542 | 0.999859665 |
| ENSG00000168209 | DDIT4 | 0.650876754 | 0.015170232 | 0.999859665 |
| ENSG00000211659 | IGLV3-25 | 1.390584389 | 0.015270047 | 0.999859665 |
| ENSG00000231327 | LINC01816 | 1.300775451 | 0.015400118 | 0.999859665 |
| ENSG00000269403 | AC008750.8 | 3.656663174 | 0.016154153 | 0.999859665 |
| ENSG00000242986 | RPL21P99 | 3.320492114 | 0.016192964 | 0.999859665 |
| ENSG00000276831 | AC020922.4 | 2.794788276 | 0.016472296 | 0.999859665 |
| ENSG00000227113 | AC073210.1 | 1.441078936 | 0.017910686 | 0.999859665 |
| ENSG00000251442 | LINC01094 | 0.631188788 | 0.018356464 | 0.999859665 |
| ENSG00000276097 | AC006538.3 | 0.957217782 | 0.01848908 | 0.999859665 |
| ENSG00000280167 | AP000943.4 | 1.620328837 | 0.018679207 | 0.999859665 |
| ENSG00000265168 | AC005726.4 | 1.325805184 | 0.018942549 | 0.999859665 |
| ENSG00000265401 | AC093484.3 | 0.798173361 | 0.019144171 | 0.999859665 |
| ENSG00000272669 | AL021707.6 | 0.672192214 | 0.020043748 | 0.999859665 |
| ENSG00000234203 | AC004771.2 | 0.741358271 | 0.021876259 | 0.999859665 |
| ENSG00000254521 | SIGLEC12 | 1.59930202 | 0.022480774 | 0.999859665 |
| ENSG00000258215 | AC078886.1 | 1.097066759 | 0.022738453 | 0.999859665 |
| ENSG00000211950 | IGHV1-24 | 1.309093595 | 0.023327616 | 0.999859665 |
| ENSG00000104524 | PYCR3 | 0.61609013 | 0.023372813 | 0.999859665 |
| ENSG00000211970 | IGHV4-61 | 2.249652366 | 0.023571355 | 0.999859665 |
| ENSG00000256072 | AC078889.1 | 1.236725622 | 0.023961223 | 0.999859665 |
| ENSG00000280064 | AC130304.1 | 0.632148955 | 0.02398568 | 0.999859665 |
| ENSG00000134463 | ECHDC3 | 0.712168575 | 0.024922781 | 0.999859665 |
| ENSG00000127946 | HIP1 | 0.617396771 | 0.025146374 | 0.999859665 |
| ENSG00000227050 | AL512288.1 | 0.963786399 | 0.025233914 | 0.999859665 |
| ENSG00000007968 | E2F2 | 0.804619904 | 0.025248702 | 0.999859665 |
| ENSG00000167900 | TK1 | 0.779732121 | 0.02567834 | 0.999859665 |
| ENSG00000185899 | TAS2R60 | 0.988323491 | 0.025689321 | 0.999859665 |
| ENSG00000258839 | MC1R | 0.670640726 | 0.02569878 | 0.999859665 |
| ENSG00000124588 | NQO2 | 0.879864844 | 0.026030491 | 0.999859665 |
| ENSG00000226823 | SUGT1P1 | 0.989772482 | 0.026333759 | 0.999859665 |
| ENSG00000013573 | DDX11 | 1.136530512 | 0.026744753 | 0.999859665 |
| ENSG00000256843 | AC023157.2 | 3.164935027 | 0.027278585 | 0.999859665 |
| ENSG00000258380 | AL356805.1 | 2.15821858 | 0.027685586 | 0.999859665 |
| ENSG00000180549 | FUT7 | 0.627320512 | 0.028631414 | 0.999859665 |
| ENSG00000072952 | MRVI1 | 0.829120421 | 0.028679626 | 0.999859665 |
| ENSG00000151726 | ACSL1 | 0.714561062 | 0.02884633 | 0.999859665 |
| ENSG00000105472 | CLEC11A | 0.748270431 | 0.029009506 | 0.999859665 |
| ENSG00000164845 | FAM86FP | 0.948491942 | 0.029834528 | 0.999859665 |
| ENSG00000232536 | AL365436.2 | 1.250113724 | 0.030039455 | 0.999859665 |
| ENSG00000270990 | AC069304.2 | 0.908634016 | 0.030441038 | 0.999859665 |
| ENSG00000275741 | AL162497.1 | 0.990372038 | 0.030807763 | 0.999859665 |
| ENSG00000229447 | AC114495.2 | 0.706403911 | 0.031261428 | 0.999859665 |
| ENSG00000237758 | BANF1P3 | 1.623330433 | 0.031773645 | 0.999859665 |
| ENSG00000007516 | BAIAP3 | 0.849547196 | 0.031806265 | 0.999859665 |
| ENSG00000238113 | LINC01410 | 0.685263295 | 0.032296277 | 0.999859665 |
| ENSG00000225439 | BOLA3-AS1 | 0.675564134 | 0.032651833 | 0.999859665 |
| ENSG00000151715 | TMEM45B | 0.608311521 | 0.032929429 | 0.999859665 |
| ENSG00000197728 | RPS26 | 1.309180898 | 0.033214022 | 0.999859665 |
| ENSG00000272056 | AC013472.3 | 0.900759831 | 0.033237154 | 0.999859665 |
| ENSG00000232645 | LINC01431 | 0.825238235 | 0.033639152 | 0.999859665 |
| ENSG00000237836 | PHKA2-AS1 | 0.930211625 | 0.033690309 | 0.999859665 |
| ENSG00000283566 | AC243725.1 | 2.108065765 | 0.034385609 | 0.999859665 |
| ENSG00000258378 | AL139021.1 | 1.929700441 | 0.034391613 | 0.999859665 |
| ENSG00000228218 | ATF4P3 | 0.742949739 | 0.034485895 | 0.999859665 |
| ENSG00000236956 | NF1P8 | 1.617113357 | 0.03473476 | 0.999859665 |
| ENSG00000251450 | RASGRF2-AS1 | 1.154411533 | 0.035356774 | 0.999859665 |
| ENSG00000099251 | HSD17B7P2 | 0.767308763 | 0.03567959 | 0.999859665 |
| ENSG00000233673 | ANAPC1P1 | 0.626979592 | 0.037128435 | 0.999859665 |
| ENSG00000258810 | AL133371.2 | 0.588693577 | 0.037634325 | 0.999859665 |
| ENSG00000060140 | STYK1 | 0.855549931 | 0.037783959 | 0.999859665 |
| ENSG00000145911 | N4BP3 | 0.601138723 | 0.038128713 | 0.999859665 |
| ENSG00000180509 | KCNE1 | 0.641858841 | 0.038497249 | 0.999859665 |
| ENSG00000229539 | AL353194.1 | 0.890202907 | 0.039076411 | 0.999859665 |
| ENSG00000251652 | AC092535.3 | 0.941962331 | 0.039356171 | 0.999859665 |
| ENSG00000170315 | UBB | 0.654826829 | 0.039512458 | 0.999859665 |
| ENSG00000270133 | AC025766.1 | 0.915927362 | 0.03966978 | 0.999859665 |
| ENSG00000258749 | AL110504.1 | 0.633838068 | 0.040088076 | 0.999859665 |
| ENSG00000223609 | HBD | 0.950290463 | 0.040676163 | 0.999859665 |
| ENSG00000160307 | S100B | 1.849142967 | 0.041286005 | 0.999859665 |
| ENSG00000171451 | DSEL | 0.951853271 | 0.04149801 | 0.999859665 |
| ENSG00000256751 | PLBD1-AS1 | 0.686121299 | 0.041762216 | 0.999859665 |
| ENSG00000145423 | SFRP2 | 1.006675783 | 0.041767699 | 0.999859665 |
| ENSG00000182885 | ADGRG3 | 0.91386673 | 0.042580316 | 0.999859665 |
| ENSG00000171236 | LRG1 | 0.753386405 | 0.043265164 | 0.999859665 |
| ENSG00000255122 | AC068587.2 | 1.441541463 | 0.043425532 | 0.999859665 |
| ENSG00000273090 | AC007378.1 | 1.324114534 | 0.04432489 | 0.999859665 |
| ENSG00000223375 | AC096638.1 | 2.913872886 | 0.044371377 | 0.999859665 |
| ENSG00000185052 | SLC24A3 | 0.589114859 | 0.044627733 | 0.999859665 |
| ENSG00000251179 | TMEM92-AS1 | 0.881506022 | 0.044883753 | 0.999859665 |
| ENSG00000177337 | DLGAP1-AS1 | 0.608252253 | 0.044904869 | 0.999859665 |
| ENSG00000166793 | YPEL4 | 0.58706096 | 0.045416384 | 0.999859665 |
| ENSG00000261471 | AC092145.1 | 0.733933366 | 0.045558346 | 0.999859665 |
| ENSG00000267232 | AC012615.5 | 1.373311293 | 0.045965318 | 0.999859665 |
| ENSG00000177854 | TMEM187 | 0.661236792 | 0.046101293 | 0.999859665 |
| ENSG00000173585 | CCR9 | 0.917514081 | 0.046670019 | 0.999859665 |
| ENSG00000220349 | Z85996.1 | 1.58221643 | 0.046899522 | 0.999859665 |
| ENSG00000178814 | OPLAH | 0.846014501 | 0.047103784 | 0.999859665 |
| ENSG00000256007 | ARAP1-AS1 | 0.637505676 | 0.047270279 | 0.999859665 |
| ENSG00000264304 | AC024267.3 | 0.932239736 | 0.047772017 | 0.999859665 |
| ENSG00000259772 | AC012236.1 | 1.319051002 | 0.049614892 | 0.999859665 |
| ENSG00000254114 | HMGN1P28 | 0.921768334 | 0.049750218 | 0.999859665 |
| ENSG00000223551 | TMSB4XP4 | 0.675056261 | 0.049868534 | 0.999859665 |
| ENSG00000211640 | IGLV6-57 | 1.301850485 | 0.04997558 | 0.999859665 |
